# Combination of Averaged Bregma-Interaural and Electrophysiology-Guided Technique Improves Subthalamic Nucleus Targeting Accuracy in Rats

**DOI:** 10.1523/ENEURO.0244-25.2025

**Published:** 2025-12-12

**Authors:** Zhengdao Deng, Ali Awada, Ugur Kilic, Myles Mc Laughlin, Christelle Baunez, Bart Nuttin

**Affiliations:** ^1^ Experimental Neurosurgery and Neuroanatomy, Department of Neurosciences, KU Leuven, Leuven 3000, Belgium; ^2^Institut de Neurosciences de La Timone, UMR 7289 CNRS & Aix-Marseille Université, Marseille 13005, France; ^3^ Exp ORL, Department of Neurosciences, The Leuven Brain Institute, KU Leuven, Leuven 3000, Belgium

**Keywords:** bregma, electrode implantation, electrophysiology, interaural, stereotactic surgery, subthalamic nucleus

## Abstract

Accurate electrode implantation in the subthalamic nucleus (STN) of rats is essential for high-quality electrophysiological and neuromodulation studies but remains technically challenging due to the small size and deep location of the STN. Traditional stereotactic methods, relying on bregma or averaged bregma-interaural-based coordinates, often result in misplacement of electrode. Here, we introduce a combined anatomical and functional approach—bregma-interaural and electrophysiology-guided technique (BITE)—designed to enhance targeting accuracy for STN electrode implantation in male Sprague Dawley rats. In this method, anterior-posterior (AP), medial-lateral (ML), and dorsal-ventral (DV) coordinates are initially determined using the average of bregma and interaural references. Electrode depth (DV axis) is fine-tuned based on real-time detection of characteristic STN neuronal firing patterns. If STN featured activity is not observed on the first implantation, additional adjustments in the AP and ML axes are made, followed by electrophysiology-guided DV tuning. Using BITE, we achieved an 83% overall success rate for STN electrode implantation, with 50% of electrodes precisely located in the dorsal STN (dSTN). This represents a significant improvement compared with the bregma-based method (17%, *p* = 0.0005) and the averaged bregma-interaural-based method (40%, *p* = 0.0188). BITE offers two main advantages: (1) increased accuracy in targeting the STN and (2) improved access to the dSTN, a region of growing interest in basal ganglia research. These findings demonstrate that BITE is a reliable and effective method for precise electrode placement in the STN and may serve as a valuable tool in rat models of deep brain stimulation and basal ganglia function.

## Significance Statement

This study introduces BITE, a standardized method that combines anatomical referencing with electrophysiological confirmation to improve electrode implantation accuracy in the rat subthalamic nucleus (STN). By averaging bregma-interaural coordinates, BITE minimizes the impact of bregma misidentification on implantation accuracy, while real-time STN firing patterns guide fine adjustments and confirm electrode placement. The method includes a rescue strategy for failed initial implantation, significantly improving overall success. BITE achieved an 83% implantation success rate, with 50% of electrodes precisely placed in the dorsal STN (dSTN). This approach shows strong potential for accurate targeting of dSTN and may be adaptable for broader applications in rat brain surgeries. Further validation is needed to confirm its utility across other brain regions.

## Introduction

The accuracy of rat stereotactic surgery is heavily dependent on the stereotaxic coordinates referenced from Paxinos and Watson's “Rat Brain in Stereotaxic Coordinates” (George Paxinos, 2007). However, unlike clinical stereotactic surgery, computed tomography (CT) and magnetic resonance imaging (MRI) are rarely employed for preoperative planning and intraoperative adjustment of electrode positioning in rats, primarily owing to certain technical challenges when applied to small animals (Rangarajan et al., 2016). As a result, the accuracy of electrode implantation in rats might be compromised. Additionally, incorrect skull flat positioning, improper insertion of the ear bars, inaccurate identification of bregma, and electrode deviation from planned trajectory during electrode implantation can remarkably diminish the implantation accuracy. Thus, precise electrode implantation in rats may pose a challenge for some researchers, potentially resulting in a substantial increase in rat use, time, energy and lab consumables expended on experiments, and even experiment failure.

The bregma-based approach has traditionally been the most widely employed method for stereotactic surgery in rats (De Vloo and Nuttin, 2019). However, reliance on this single-reference system presents two main disadvantages: (1) bregma identification presents a persistent challenge in stereotaxic surgery due to anatomical variability, frequently leading to localization errors. Such inaccuracies can substantially compromise the accuracy of electrode implantation. (2) The bregma-based approach may inherently yield reduced electrode implantation accuracy compared with dual-reference system (Paxinos et al., 1985). To address this issue, some studies have adopted averaged coordinates calculated between bregma-based and the interaural-based coordinates (Baunez and Robbins, 1997; Faust et al., 2021). However, the accuracy of averaged bregma-interaural-based approach remains vulnerable to significant inaccuracy when bregma is misidentified due to anatomical variability. Thus, a new approach capable of addressing these limitations while achieving optimal targeting accuracy is warranted.

Electrophysiology has been regularly applied in electrode implantation for patients (Rijks et al., 2022; Nho et al., 2024). However, its application for electrode implantation in rats remains uncommon (Maesawa et al., 2004; Fluri et al., 2015). Current electrophysiology-guided electrode implantation conventionally relies on bregma-based approach. However, the bregma-based approach has its disadvantages, as discussed previously.

In the present study, we used a hybrid methodology, combining averaged bregma-interaural-based approach and electrophysiology-guided technique (BITE) and, if necessary, multiple trajectories to realize precise electrode implantation in rats. Strikingly, BITE mitigates the influence of skull anatomical variability on the accuracy of electrode implantation. To evaluate BITE accuracy, we chose subthalamic nucleus (STN) as the implantation site due to four reasons: (1) The STN in rats presents significant implantation challenges due to its small dimensions. It measures anterior-posteriorly 1.2 mm, 0.8 ± 0.1 mm (De Vloo and Nuttin, 2019), and is situated deeply 7.4–8.8 mm beneath the skull surface (Hardman et al., 2002; George Paxinos, 2007). (2) STN is a “hot-spot” structure in various fields including neuromodulation, movement disorders, and some psychiatric disorders, (3) the averaged bregma-interaural-based approach has been previously used for STN targeting and proven relatively efficient (Baunez and Robbins, 1997; Baunez et al., 2001), and (4) electrophysiological characterization can reliably identify the STN based on its distinctive firing pattern (Maesawa et al., 2004; Deng et al., 2025).

In this study, we first conducted a retrospective analysis evaluating the targeting success rates of bregma-based approach and the averaged bregma-interaural-based approach. We then prospectively evaluated the targeting success rates of BITE, using STN electrode implantation as our model procedure. Our specific objectives were to (1) evaluate the accuracy of BITE, (2) quantitatively compare the implantation accuracy across all three targeting approaches, and (3) propose a strategy for adjusting the electrode positioning to increase implantation accuracy.

## Materials and Methods

### Subjects

Experiments included 80 male Sprague Dawley rats purchased from Charles River Laboratories. All procedures were conducted in accordance with an approved ethical protocol (P091/2021) and complied with national legislation (Royal Decree regarding the protection of laboratory animals of 29 May 2013) and European directive 2010/63/EU.

### Experiment design

This study comprised three experiments: a retrospective study (Experiment 1) and two prospective studies (Experiments 2 and 3). All stereotaxic coordinates for STN were referenced to the Paxinos and Watson atlas (George Paxinos, 2007). To control potential confounders when comparing targeting success rates across approaches, (1) we used rats of identical strain with tightly regulated body weights. A further statistical analysis comparing the body weights are provided below. (2) All procedures employed the same stereotaxic apparatus with consistent precision. (3) A single experienced researcher performed all electrode implantations to eliminate variability from surgical expertise. (4) The operator rigorously followed established protocols for each approach without modification or optimization, ensuring methodological consistency throughout the study to reduce the learning curve effect. A further statistical analysis evaluating the learning curve effect are provided below. Specifically, the procedure for the bregma-based approach is outlined below from Steps 1.1 to 1.5, excluding Step 1.4. The averaged bregma-interaural-based approach is described from Steps 1.1 to 1.6, while the electrode implantation in the STN using BITE is detailed from Steps 1.1 to 1.8.

#### Experiment 1

We aimed to retrospectively evaluate the targeting success rates of traditional approaches: the bregma-based approach and the averaged bregma-interaural-based approach. All of 58 rats that underwent electrode implantation between 2022 and 2023 were included in the analysis, with 18 rats receiving the bregma-based approach and 40 rats receiving the averaged bregma-interaural-based approach. The targeting accuracy of both approaches was histologically verified through hematoxylin and eosin (H&E) staining to confirm electrode tip position.

#### Experiment 2 (*n* = 10)

We aimed to evaluate the anterior-posterior (AP) deviation of electrode implantation caused by averaged bregma-interaural-based approach. This knowledge helps us to adjust the electrode position in Experiment 3. To achieve this goal, we verified the exact electrode implantation sites using H&E staining and quantified the AP deviation by measuring the AP distance between the actual implantation site and the intended implantation site (3.7 mm posterior to bregma). The actual implantation site in AP axis was determined by reference to the Paxinos and Watson atlas (George Paxinos, 2007). The evaluation of dorsal-ventral (DV) and medial-lateral (ML) deviations of electrode implantation caused by averaged bregma-interaural-based approach were not evaluated in this study. The reasons are as follows: (1) the skull was removed, making it impossible to accurately measure the DV length between the electrode tip and the skull surface based on the histological results. Furthermore, precise electrode implantation of STN was achieved using electrophysiology rather than DV coordinate in this study. (2) The accuracy of averaged bregma-interaural-based approach in ML axis is supposed to be relatively high, resulting in minimal ML deviation. This will be discussed further in the Discussion.

#### Experiment 3 (*n* = 12)

We aimed to prospectively evaluate the accuracy of BITE. To achieve this goal, we executed electrode implantation of STN using BITE. The accuracy of BITE was histologically verified through H&E staining to confirm electrode tip position.

A 69100 rotational digital stereotaxic frame for rat (RWD Life Science) was used for performing stereotactic surgery in this study. Microelectrodes (*ϕ* = 125 μm, FHC) composed of platinum/iridium (200–300 kΩ) were used for neural recording. The data of extracellular electrophysiology was acquired through an Open Ephys acquisition board and was real-time virtualized through Open Ephys GUI (Siegle et al., 2017).

### Standardized protocol for electrode implantation in STN

#### Step 1.1: Anesthesia and surgical preparation

Rat body weight was measured and recorded before surgery. The anesthetic solution was a mixture of 0.6 ml ketamine (100 mg/ml, Nimatek, Dechra Pharmaceuticals) and 0.4 ml medetomidine (1 mg/ml, Domitor, Orion Corporation). Surgical procedures were conducted under deep anesthesia through a peritoneal injection of the mixture at the dosage of 0.1 ml/100 mg body weight. Anesthesia was maintained by administering 0.1 ml of the mixture per hour via peritoneal injection. Body temperature was kept constant at 37 ± 0.5°C through a ThermoStar Homeothermic Monitoring System (RWD Life Science). Ophthalmic ointment (Duratears, Alcon) was applied to both eyes to protect ocular surface.

#### Step 1.2: Achievement of ideal head fixation on the stereotactic apparatus

##### Head fixation

A 2% Xylocaine gel (Aspen Pharma Trading) was applied onto the ear bar tips. Beginning with the left ear bar positioned on the apparatus (we used a right-handed operator as an example), we carefully inserted the ear bar into the left ear canal of the rat. Next, the right ear bar was inserted into the right ear canal. Both ear bars were then adjusted along the ML axis by hands until the scales were identical on both sides.

##### Head stability test

**Figure 1.**
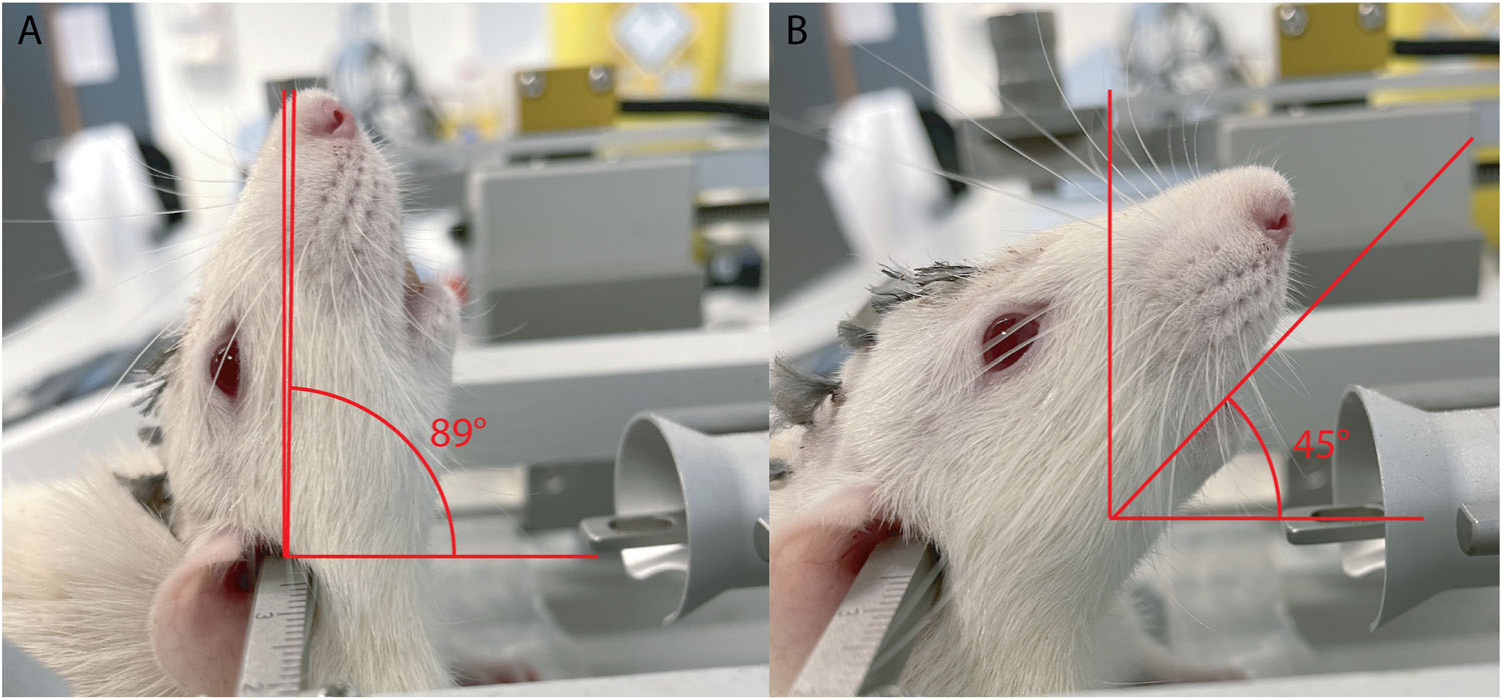
Head stability test. ***A***, The rat's head is gently tilted upwards to ∼90° (in the case of this figure 89°), no sign of droop. ***B***, Rat's head is in 45°, no sign of droop. The *x-*axis is horizontal line parallel to the ground floor.

After securing the rat’s head in position, head stability test was executed. First, the rat’s head was gently tilted upwards to ∼90° (Fig. 1*A*). If the head remained stable without any sign of droop, we then gently pushed the head forward to ∼45° (Fig. 1*B*). During this movement, resistance was felt, and the head showed no signs of droop when this motion was topped before reaching 45°. Throughout this entire head motion, the rat’s head pivoted on the ear bars. Successfully maintaining in these two angles for 10 s, with resistance and no signs of droop immediately after a sudden stop during the head pivoting, confirmed that the rat’s head was perfectly fixed on the stereotactic apparatus.

##### Identification of bregma

**Figure 2.**
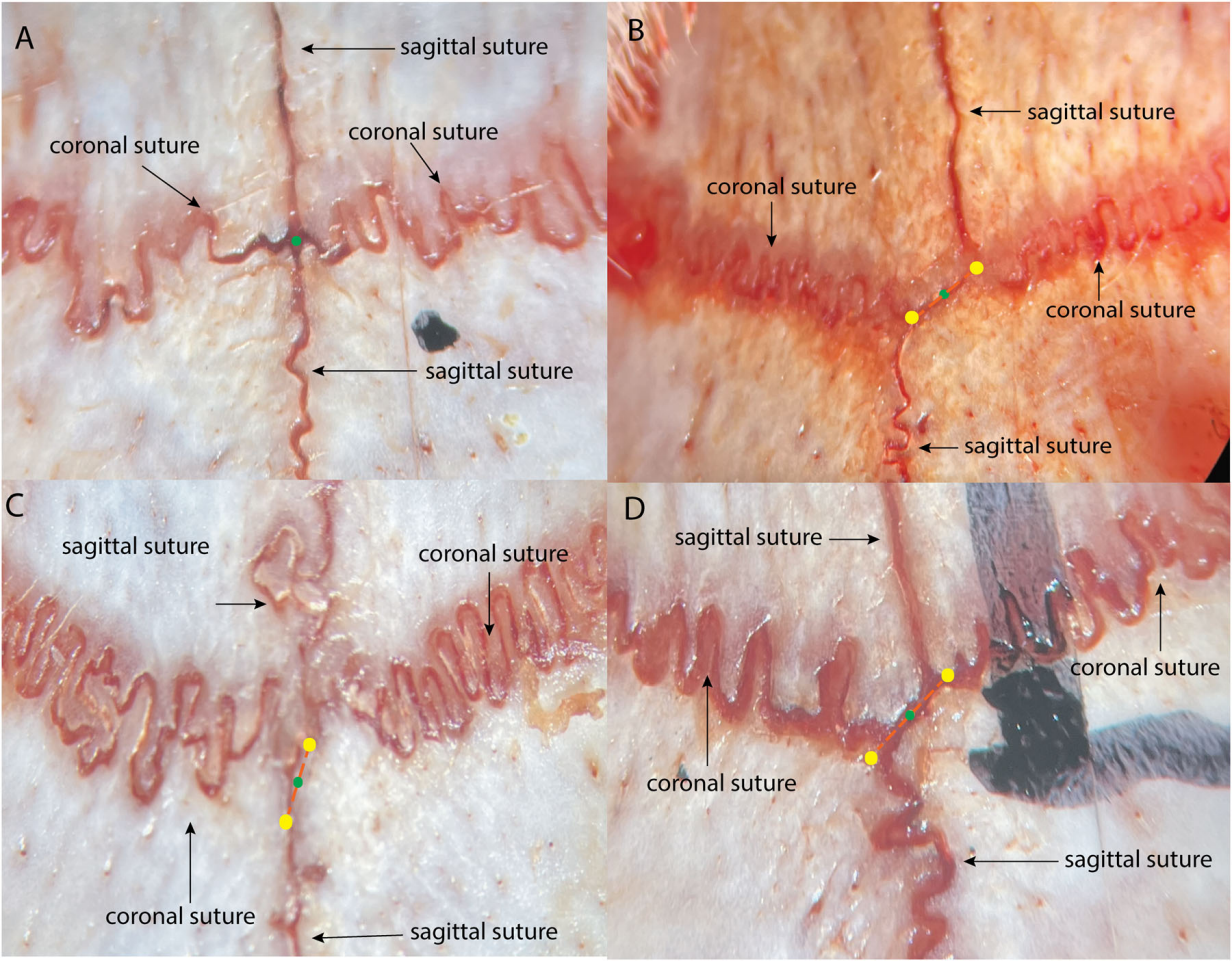
Identification of bregma. ***A***, Bregma is marked by a green point where the sagittal suture and coronal suture meet. ***B–D***, Anatomical variability results in two intersection points between the sagittal and coronal sutures. The bregma, marked by a green point, is identified as the midpoint between the two intersections, which are marked by yellow points.

After exposing the skull, we located bregma according to two criteria: (1) if the sagittal and coronal sutures intersected at a single point, bregma was defined at that intersection point (Fig. 2*A*). (2) If skull anatomical variability resulted in two intersection points between the sagittal and coronal sutures (Fig. 2*B–D*), bregma was defined as the midpoint between these two intersections.

##### Verification of ideal flat skull position

**Figure 3.**
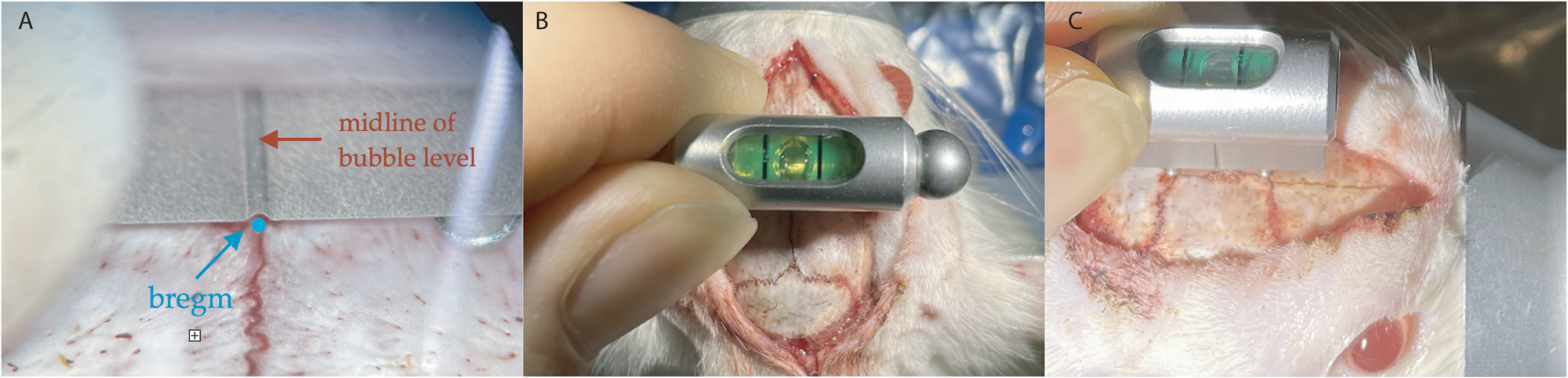
Verification of ideal flat skull position. ***A***, The bubble level is positioned in ML axis. Bregma is marked by a blue point. The bubble level's metal plate features a central indentation, which is carefully aligned with the marked bregma during positioning. ***B***, The bubble level is positioned in ML axis. The bubble rests at the center point, indicated by two black vertical lines, when the ideal flat skull position in ML axis is achieved. ***C***, The bubble level is positioned in AP axis. The bubble rests at the center point when the ideal flat skull position in AP axis is achieved. Please note that the absence of gloves is for demonstration purposes only. All procedures were performed in accordance with standard safety and hygiene protocols.

BITE is based on the atlas, making the attainment of an ideal flat skull position crucial (George Paxinos, 2007). This process involves two steps: (1) verification of ideal flat skull position in ML axis: a bubble level probe (Invilog Research) was positioned on the skull in ML axis, aligning the midline of the bubble level with bregma (Fig. 3*A*). The ideal flat skull position in the ML axis was achieved when the bubble rested at the center point (Fig. 3*B*). (2) Verification of ideal flat skull position in AP axis: a bubble level probe was positioned on the skull in AP axis. The height of the tooth bar was finely adjusted until the bubble rested at the center point (Fig. 3*C*).

#### Step 1.3: Pinpointing the projection of STN on the skull and skull drilling

We marked the approximate location of the STN projection on the skull (3.7 mm posterior and 2.4 mm lateral to bregma) using the bregma-based approach, and a burr hole was then created, large enough to facilitate electrode implantation through this cranial opening.

The skull drilling process generates high-frequency vibrations and mechanical forces. We hypothesize that these forces could cause minor shift of the skull, potentially leading to a bregma shift. Although we have not yet quantified this phenomenon, we occasionally observed indications of such shifts during surgery. Thus, we recommend that operators perform the critical step of coordinate calculation (Step 1.5) after any major drilling to preemptively mitigate this potential source of error. Future work is needed to quantify this phenomenon.

#### Step 1.4: Calculations of STN coordinates based on interaural zero

To ensure the accuracy of STN coordinates, we removed the rat's head from the stereotactic apparatus to determine the STN coordinates based on the interaural zero. Afterward, we securely refixed the rat's head to the stereotactic apparatus and determined the STN coordinates using bregma.

**Figure 4.**
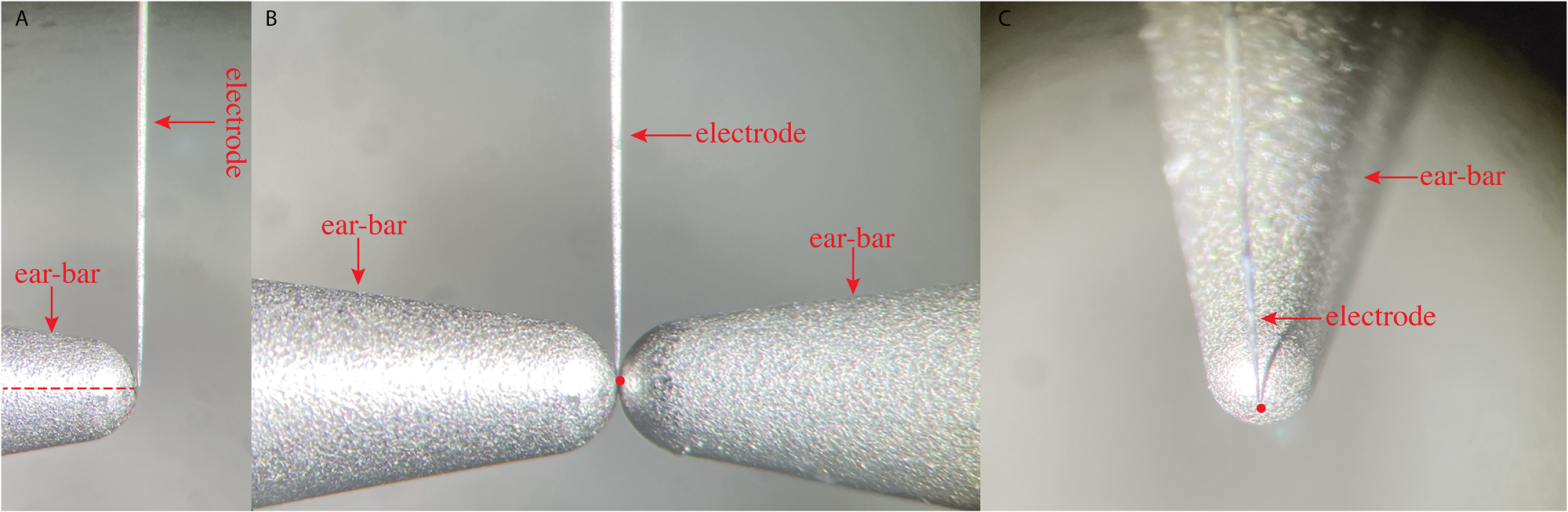
Determine the coordinates of interaural zero. ***A***, Determining the dorsal-ventral coordinate of interaural zero. The red dotted line represents the axis of the ear bar. The electrode tip is precisely aligned with the ear bar tip. ***B***, Determining the medial-lateral coordinate of interaural zero. The red dot represents the meeting point of two ear bars. The electrode tip is well positioned over the meeting point of two ear bars. ***C***, Determining the anterior-posterior coordinate of interaural zero. The red dot represents the ear bar tip. The electrode tip is aligned to the ear bar tip.

Using a micromanipulator and surgical microscope, we adjusted the electrode position until the tip was precisely aligned with the ear bar tip (Fig. 4*A–C*), the scales on the DV, ML, and AP arms were then recorded as AP’, ML’, and DV’, representing the coordinates of the interaural zero. The STN coordinates based on interaural zero were measured as 5.3 mm anterior (AP_interaural_ = AP’ + 5.3), 2.4 mm lateral for the right STN (ML_interaural_ = ML’ − 2.4), and 1.65 mm superior (DV_interaural_ = DV’ + 1.65) to interaural zero, respectively (George Paxinos, 2007).

#### Step 1.5: Calculations of STN coordinates based on bregma

Using a surgical microscope, we carefully aligned the electrode as close as possible to the skull surface at bregma and recorded the scales as AP’, ML’, and DV’ on the respective arms. According to the atlas (George Paxinos, 2007) and the previous experiences in targeting STN (Baunez and Robbins, 1997; Baunez et al., 2001; Winstanley et al., 2005; Siegle et al., 2017; Pelloux et al., 2018), the STN coordinates relative to bregma were determined as 3.7 mm posterior (AP_bregma_ *=* AP’ − 3.7), 2.4 mm lateral for the right STN (ML_bregma_ *=* ML’ − 2.4), and 8.35 mm ventral (DV_bregma_ = DV − 8.35).

#### Step 1.6: Calculations of STN coordinates based on averaged bregma-interaural-based approach

We calculated the absolute values of difference between bregma-based and interaural-based STN coordinates as |AP_bregma_ − AP_interaural_|, |ML_bregma_ − ML_interaural_|, and |DV_bregma_ − DV_interaural_|. The difference thresholds were set to |AP_bregma_ − AP_interaural_| ≤ 0.5 mm, |ML_bregma_ − ML_interaural_| ≤ 0.3 mm, and |DV_bregma_ − DV_interaural_| ≤ 0.3 mm. If the calculated absolute values of difference were within the difference thresholds, we averaged the bregma-based and interaural zero-based coordinates to obtain the final STN coordinates: AP_bregma*-*interaural_ = (AP_bregma_ − AP_interaural_) / 2, ML_bregma*-*interaural_ = (ML_bregma_ − ML_interaural_) / 2, and DV_bregma*-*interaural_ = (DV_bregma_ − DV_interaural_) / 2. If any differences exceeded the difference thresholds, we used the bregma-based coordinates for AP, ML, or DV.

#### Step 1.7: Determination of precise STN DV position using electrophysiology

**Figure 5.**
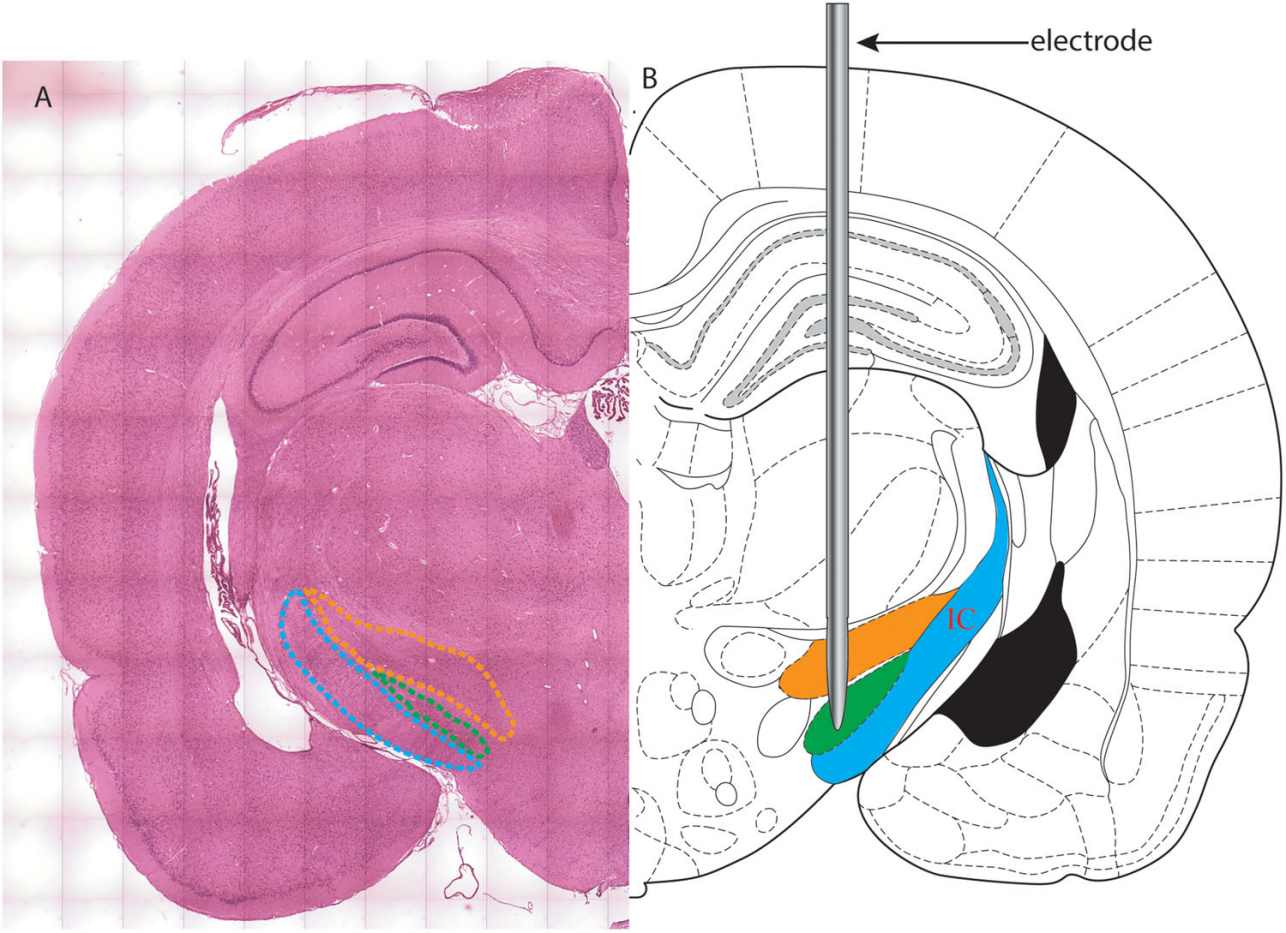
Schematic diagram of a coronal section of the rat brain. The zona incerta (ZI) is located dorsal to the subthalamic nucleus (STN), which in turn lies dorsal to the internal capsule (IC). ***A***, A coronal section of a rat brain stained with hematoxylin and eosin. The STN is highlighted with a green dotted line, while the ZI is highlighted with a yellow dotted line. The IC is highlighted in blue dotted line. ***B***, An electrode is implanted in STN, which is marked by the green area. The zona incerta (ZI) is highlighted in yellow, and the IC is highlighted in blue.

We carefully removed the dura using a needle through the cranial opening. The electrode was then lowered down until it reached the position 1 mm above DV_bregma-interaural_. We slowed down the implantation speed (∼0.02–0.05 mm/s) while closely observing the electrophysiological recording. The electrode typically entered zona incerta (ZI) before reaching STN (Fig. 5). The electrophysiology of ZI is characterized by near-silent neural activity (Maesawa et al., 2004). The electrophysiological characteristics of STN are distinctive, exhibiting a burst-firing pattern with high-amplitude (0.5–1 μV), fast-bursty, and irregular spikes (Maesawa et al., 2004; Deng et al., 2025). Upon observing the STN-like firing pattern, we immediately stopped implantation and cemented the electrode in place on the skull using four screws which were inserted before making the burr hole.

#### Step 1.8: Strategy for adjusting electrode position

Adjustment of the electrode position was applied if STN-like firing pattern was not detected 1 mm above and 0.1 mm below DV_bregma-interaural_. We slowly took out the electrode and manipulated it 0.5 mm posteriorly to the AP_bregma-interaural_ and repeated Step 1.7. If the STN-like firing pattern was still not observed, we subsequently planned maximally five additional implantations based on the coordinates provided below.

0.5 mm posterior to AP_bregma-interaural_ and 0.2 mm medial to AP_bregma-interaural_.

0.5 mm posterior to AP_bregma-interaural_ and 0.2 mm lateral to AP_bregma-interaural_.

0.5 mm anterior to AP_bregma-interaural_.

0.5 mm anterior to AP_bregma-interaural_ and 0.2 mm medial to AP_bregma-interaural_.

0.5 mm anterior to AP_bregma-interaural_ and 0.2 mm lateral to AP_bregma-interaural_.

If STN-like firing pattern was still not observed in this step, the electrode was ultimately implanted based on averaged bregma-interaural-based coordinates.

##### Verification of electrode tip position

The electrode tip position was confirmed through histological analysis. Following perfusion with phosphate-buffered saline and 4% paraformaldehyde, the brain was extracted, embedded in paraffin, and coronally sectioned at a thickness of 10 µm. Every section containing the visible electrode track was collected and stained with H&E. The precise location of the electrode tip was determined by aligning the histological sections with the corresponding sections in the Paxinos and Watson rat brain atlas (George Paxinos, 2007). This alignment was performed using some anatomical landmarks visible in both the H&E-stained sections and the atlas, including the third ventricles and the shape of hippocampus, optic fiber. Additionally, the H&E-stained STN is in dark purple with densely packed neurons and is clearly delineated from the surrounding zona incerta and internal capsule (Fig. 5). These features facilitate the precise determination of the electrode tip location within STN when implantation is successful. An implantation of the electrode was considered a “success” if the electrode tip was located anywhere within the anatomical boundaries of the STN, regardless of whether it matched the intended coordinates. The AP coordinate of the electrode tip was obtained from the atlas (George Paxinos, 2007). In Experiment 2, this AP coordinate was used to calculate the AP deviation as the absolute difference between the actual and intended coordinates.

##### Power spectral density

We aimed to investigate the STN oscillations. To achieve this, we analyzed STN oscillations using spectral analysis. Raw data were collected at 30 kHz. Power spectral density was computed via short-time Fourier transforms with standard parameters (Hanning window, 50% overlap). This approach followed the methodology detailed in Deng et al. (2025).

##### Firing rate calculation

Raw signals were bandpass filtered (300–3,000 Hz) to isolate spikes. Spike detection was performed using a threshold based on the median absolute deviation, followed by template matching to refine spike selection. Spike times were used to calculate the spike count and firing rate (spikes per second).

### Statistical analysis

#### Primary analysis of difference in success rates

We first verified expected cell frequencies for all contingency tables. For cells with expected counts <5, we employed Monte Carlo simulation. Otherwise, Pearson’s chi-square test was applied. If previous test reached significance (*p* < 0.05), we further conducted post hoc pairwise comparisons using either chi-square tests or Fisher’s exact tests. To account for multiple comparisons, we applied Bonferroni’s correction, adjusting the significance threshold to *α* = 0.0167 for three pairwise comparisons.

#### Effect size estimation

We calculated relative risk (RR) with 95% confidence intervals (CIs) to quantify the magnitude and direction of effects between methods.

#### Success rate dynamics

To assess potential learning curve effects, we compared early versus later success rates for bregma-based approach and averaged bregma-interaural-based approach. For the bregma-based method (2022), success rates were compared between the first and second 6 months. For the averaged bregma-interaural-based method (2023), we made the same comparison. Before analysis, expected cell frequencies in all contingency tables were checked: Fisher's exact test was used when any expected count was <5, otherwise Pearson's chi-square test was applied.

To assess whether body weight differs among rats subjected to bregma-based, bregma-interaural based, and BITE procedures, we first evaluated data normality using the Kolmogorov–Smirnov test. For normally distributed data (*p* ≥ 0.05), we assessed homogeneity of variance using Bartlett's test. If variance was homogeneous (*p* ≥ 0.05), we performed one-way ANOVA. If not (*p* < 0.05), we used Welch’s ANOVA test. For non-normal distributions (*p* < 0.05), we employed the more robust Brown–Forsythe test for variance analysis, followed by the appropriate statistical test based on the result.

All analyses were performed using Prism 9 (GraphPad Software) and SPSS version 29 (IBM).

## Results

### Body weight

**Figure 6.**
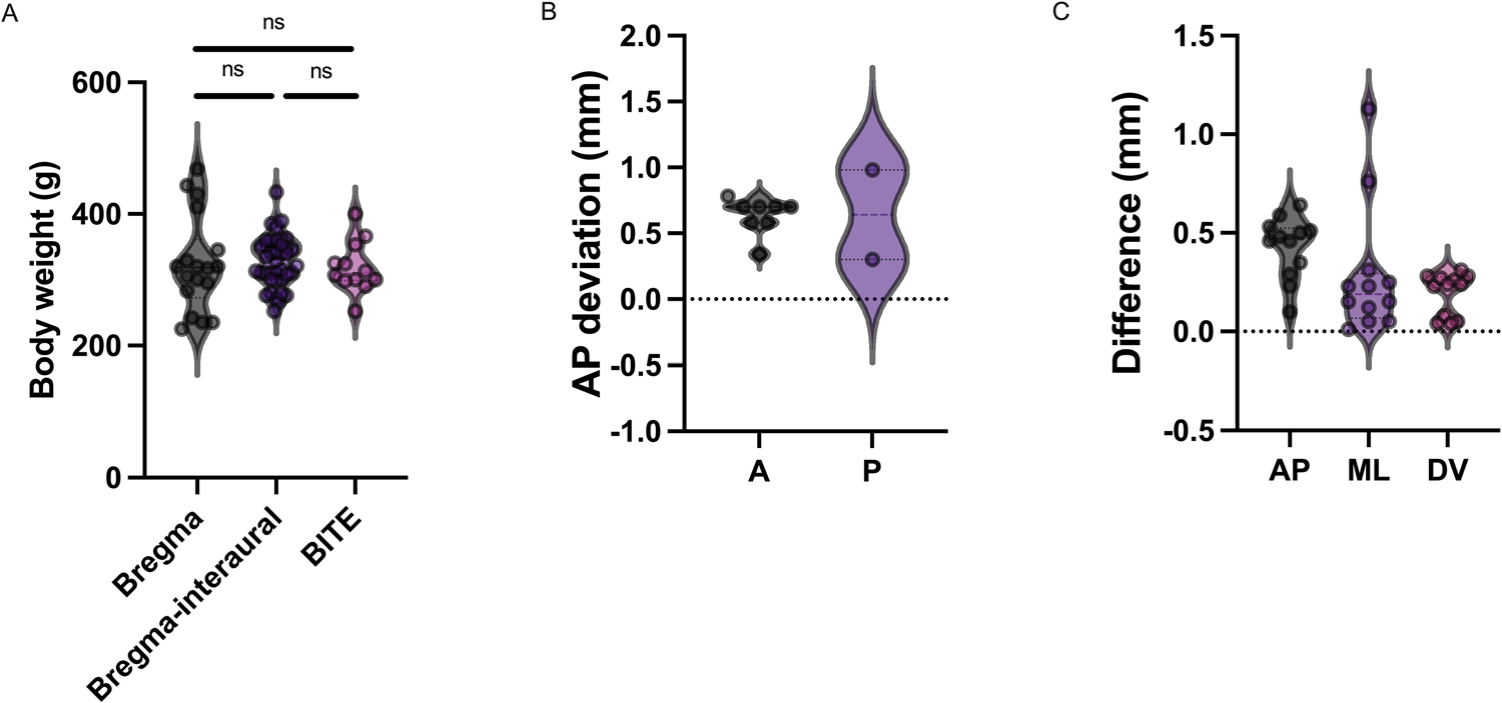
Data distribution. ***A***, Body weight distributions of rats used to evaluate the bregma-based approach, averaged bregma-interaural-based approach, and BITE are shown. No significant differences were observed among the groups (Welch's *F*_(2.00,24.57)_ = 0.2415, *p* = 0.7873). ***B***, It shows that the distribution of the anterior-posterior deviation of electrode implantation caused by averaged bregma-interaural-based approach. ***C***, It shows that the distribution of the absolute value of difference between bregma-based and interaural-based STN coordinates.

The mean body weights (±standard deviation) of the rats subjected to the bregma-based approach, the averaged bregma-interaural-based approach, and BITE were 323 ± 72 grams, 327 ± 38 grams, and 319 ± 39 grams (Fig. 6*A*), respectively, with no significant differences observed among them (Welch's *F*_(2.00,24.57)_ = 0.2415, *p* = 0.7873).

### Success rate dynamics

There was no significant difference in the success rate of the bregma-based approach between the first and second 6 month periods (0% vs 33%; *p* = 0.206; Fisher's exact test). Similarly, the success rate of the averaged bregma-interaural-based approach did not differ significantly between the first and second 6 month periods (44% vs 30%; *p* = 0.449; Fisher's exact test).

Experiment 1: Retrospective evaluation of success rate of bregma-based approach and average bregma-interaural-based approach

The success rate of bregma-based approach (*n* = 18) was 17% (3/18), while the success rate of averaged bregma-interaural-based approach (*n* = 40) was 40% (16/40).

Experiment 2: Evaluation of AP deviation of electrode implantation caused by averaged bregma-interaural-based approach

Ten rats were included in Experiment 2. The electrodes were implanted more anteriorly than the intended site in eight rats (80%, 8/10), with an average deviation of 0.635 mm. Electrodes were implanted more posteriorly than the intended site in two rats (20%, 2/10) with an average deviation of 0.640 mm (Table 1, Fig. 6*B*).

**Table 1.** Evaluation of anterior-posterior deviation of electrode implantation caused by averaged bregma-interaural approach

Rat name	Difference in distance (millimeter)	Relative location of the actual implantation site to the intended implantation site
Rat_001	0.58	A
Rat_002	0.70	A
Rat_003	0.30	P
Rat_004	0.70	A
Rat_005	0.58	A
Rat_006	0.78	A
Rat_007	0.70	A
Rat_008	0.98	P
Rat_009	0.34	A
Rat_010	0.70	A

In eight rats, the electrodes were implanted more anteriorly than intended, with an average deviation of 0.635 mm. In two rats, the electrodes were implanted more posteriorly than intended, with an average deviation of 0.640 mm. A indicates that the actual implantation site is anterior to the attempted implantation. P indiacates that the actual implantation site is posterior to the attempted implantation.

Experiment 3: Prospective evaluation of the accuracy of BITE

**Figure 7.**
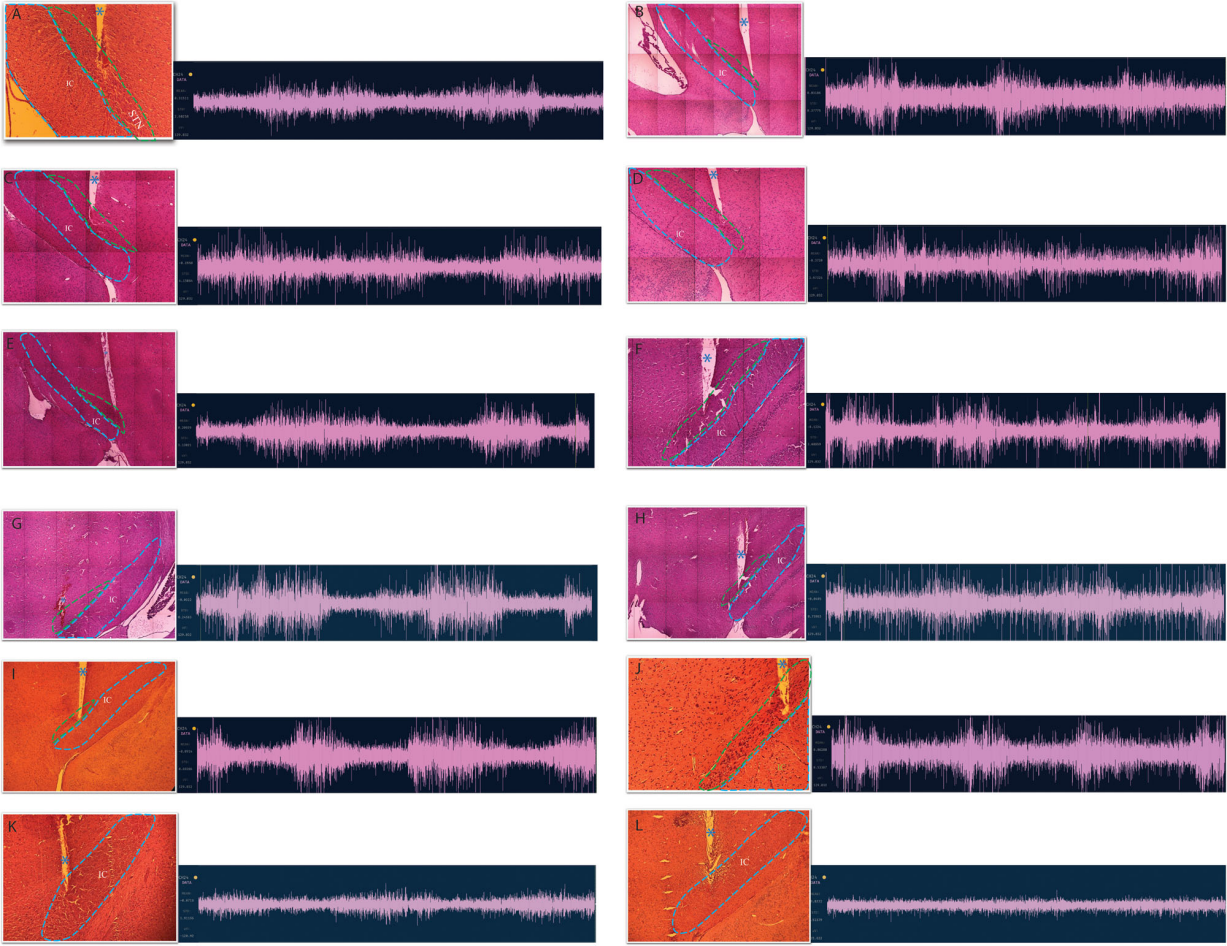
Results for histology and electrophysiological recordings. Panels ***A–L*** depict the implantation sites and electrophysiological recordings obtained from 12 rats. ***A–F***, They show the implantation sites are close to the dorsal STN. ***G–J***, They show the implantation sites are close to ventral STN. ***K–M***, The implantation sites are in internal capsule (IC). The STN is outlined with a green dotted line, and the IC is outlined with a blue dotted line. The STN firing pattern is characterized by a burst-firing pattern with high-amplitude, fast-bursty, and irregular spikes (***A–J***), while the IC firing pattern is characterized by near-silent neural activity (***K–L***). The blue asterisk indicates the electrode track.

The success rate of BITE (*n* = 12) was 83% (10/12). The dorsal region of STN (dSTN) was targeted in 6 rats (50%, 6/12; Fig. 6*A–F*). The ventral region of STN was targeted in four rats (Fig. 6*G–J*). For the two unsuccessful BITE cases, the electrodes were implanted in the internal capsule (IC; Fig. 6*K–L*). Electrode position adjustment was performed in 10 rats, with eight successful adjustments achieved (80%, 8/10). Of the successful adjustment cases, seven rats underwent a single adjustment (88%), while one rat underwent three adjustments (12%). No significant brain tissue damage was observed, except at the final electrode implantation site. The actual implantation sites of all rats were depicted in Figure 7.

### Comparison of success rate across three targeting approaches

Our primary analysis identified significant difference in success rates among the three targeting approaches (Monte Carlo *χ*²_(2)_ = 13.27, *p* = 0.0018, 95% CI: 0.001–0.003). Post hoc comparisons revealed that superior performance of BITE versus conventional approaches: (1) fivefold greater success than bregma-based targeting (83% vs 17%; *p* = 0.0005; RR = 5.0, 95%CI: 1.96–14.58, Fisher's exact test); (2) twofold greater success than bregma-interaural targeting (83% vs 40%; *p* = 0.0188; RR = 2.08, 95%CI: 1.24–3.30, Fisher's exact test). Additionally, we found no significant difference between conventional approaches (bregma-based 17% vs bregma-interaural 40%; *p* = 0.1299; RR = 0.4167, 95%CI: 0.1385–1.092, Fisher's exact test).

### The absolute value of difference between bregma-based and interaural-based STN coordinates

The absolute value of difference between bregma-based and interaural-based STN coordinates were 0.42 ± 0.16 mm (AP), 0.28 ± 0.32 mm (ML), and 0.19 ± 0.10 mm (DV), respectively (Table 2, Fig. 6*C*). Four rats used AP_bregma_ with one unsuccessful electrode implantation. Three rats used ML_bregma_, with one unsuccessful implantation. One rat used DV_bregma_, resulting in a successful implantation. In rats with unsuccessful implantation in STN, either |AP_bregma_ − AP_interaural_| or |ML_bregma_ − ML_interaural_| exceeded twice the difference threshold.

**Table 2.** The absolute value of difference between bregma-based and interaural-based STN coordinates

Rat name	AP	ML	DV	Implantation site
Rat_001	0.35	0.05	0.26	STN
Rat_002	0.29	0.12	0.08	STN
Rat_003	0.48	0.05	0.23	STN
Rat_004	0.46	0.15	0.28	STN
Rat_005	0.51	0.25	0.27	STN
Rat_006	0.23	0.01	0.24	STN
Rat_007	0.46	0.31	0.24	STN
Rat_008	0.53	0.15	0.05	STN
Rat_009	0.59	0.23	0.04	STN
Rat_010	0.50	0.23	0.31	STN
Rat_011	0.64	0.76	0.28	IC
Rat_012	0.10	1.13	0.04	IC

The absolute value of difference between bregma-based and interaural-based STN coordinates. AP, anterior-posterior; ML, medial-lateral; DV, dorsal-ventral; STN, subthalamic nucleus; IC, internal capsule.

### Electrophysiology

**Figure 8.**
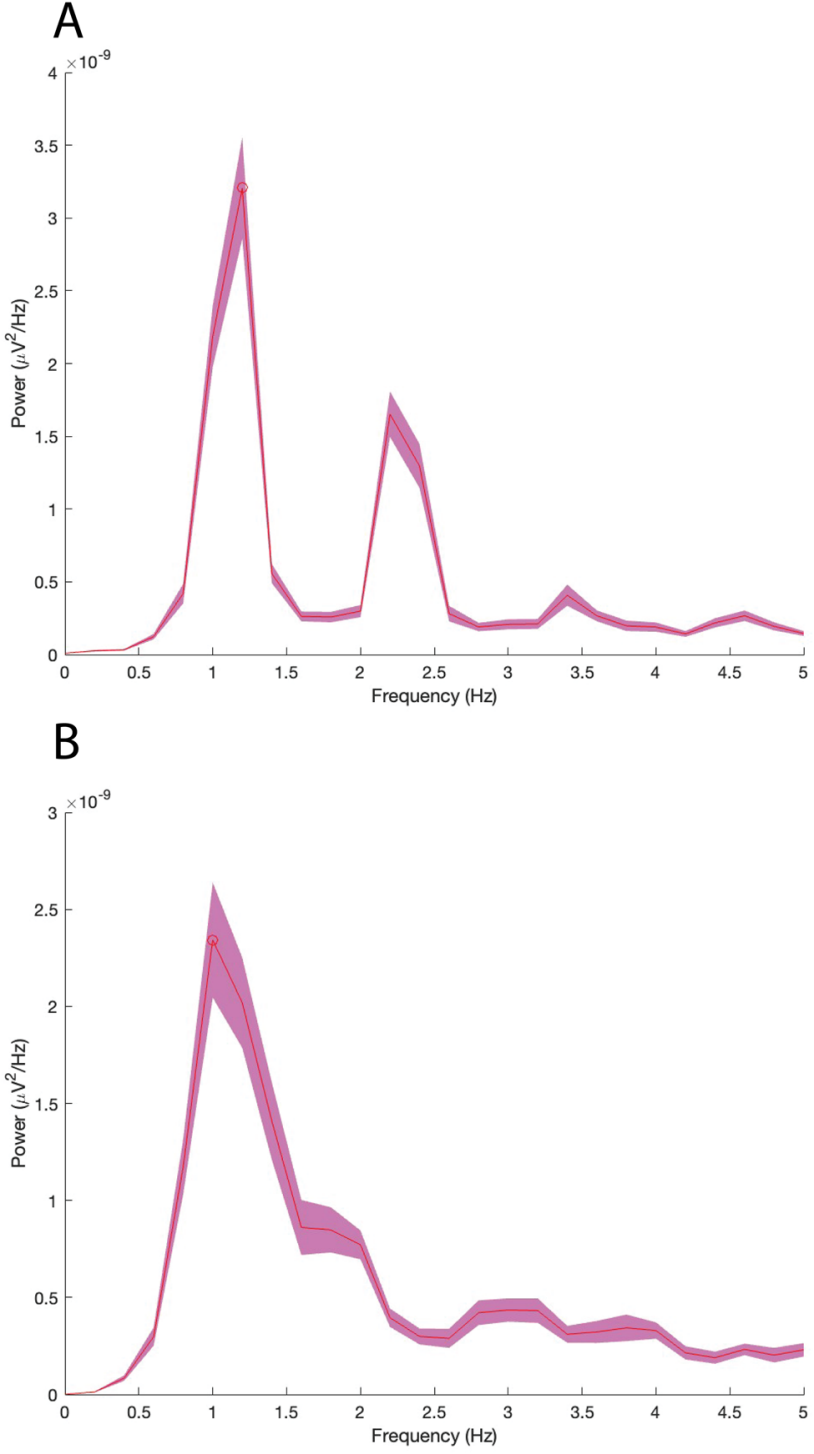
The analysis of power spectrum density. ***A***, Two peaks in the delta band are observed in the STN recording from a randomly selected rat. ***B***, One peak in the delta band is observed in the ZI recording from a randomly selected rat. STN, subthalamic nucleus. ZI, zona incerta.

The average STN firing rate was 51 ± 8 spikes per second (spk/s). STN firing pattern was characterized as a burst-firing pattern with high-amplitude (0.5–1 μV), fast-bursty, and irregular spikes (Fig. 7*A–J*). Power spectral density analysis revealed that the observed peaks were confined to delta frequency band range (0.5–4.5 Hz). Notably, among the 10 rats with successful electrode implantation, seven rats displayed two peaks in delta band (Fig. 8*A*), while the remaining three rats exhibited three peaks in the same band. The frequencies of the first, second, and third peaks were 0.6 ± 0.5 Hz, 2.0 ± 0.8 Hz, and 2.2 ± 0.4, respectively. In contrast, two rats with electrode misplacement in IC exhibited only a single peak in delta band (Fig. 8*B*). IC firing pattern was characterized as relatively low-amplitude firing without any fast-bursty firings (Fig. 7*K–L*).

## Discussion

In this study, we developed a standardized protocol for precise electrode implantation in the rat STN using BITE, assessed its targeting accuracy, and compared success rates across three different approaches. Our findings demonstrate that BITE has superior performance than traditional approaches in STN implantation. Additionally, BITE achieves a success rate of 83% for STN electrode implantation, which is comparable with or even higher than other methods (Fluri et al., 2015; Huotarinen et al., 2019), while offering the following additional advantages: (1) BITE effectively mitigates the impact of skull anatomical variability on the accuracy of electrode implantation. (2) Electrophysiology is used as an indicator, visually confirming whether the electrode is in STN and guiding fine-tuned adjustment of the electrode implantation if necessary. (3) The strategy for adjusting electrode position serves as an effective rescue approach if the first implantation is unsuccessful. Importantly, BITE demonstrates significant promise in two aspects: (1) BITE may enable accurate electrode implantation into dSTN. In this study, 50% of the electrodes were in dSTN, underscoring the method's efficacy for this application. (2) BITE holds promise as a generic approach for electrode implantation in rat brain. However, further validation is required before its generic application. The main concern for its generic application is its reliance on electrophysiological features, rendering it unsuitable for some brain structures lacking electrophysiological features (e.g., white matter). Consequently, BITE is generally not advised for electrode implantation in these regions. However, most research focuses on functional brain structures (e.g., some specific nuclei in the basal ganglia, thalamus, and cerebellum), which typically exhibit discernible electrophysiological signatures. These functional nuclei remain promising targets for BITE. Importantly, adapting BITE for electrode implantation in other electrophysiologically active brain structures is theoretically feasible, which may offer high success rate in electrode implantation.

### Skull anatomical variability and bregma identification

Paxinos stated in the atlas (George Paxinos, 2007) that “bregma is the midpoint of the curve of best fit along the coronal suture” (Rangarajan et al., 2016). However, accurately identifying this cranial landmark presents challenges and may lead to misidentification of bregma: (1) the curve of best fit along the coronal suture was not mathematically specified by Paxinos, making it difficult to determine precisely. (2) Pinpointing the midpoint of this curve adds further complexity Blasiak et al., 2010). To identify bregma, we proposed the criteria. However, these criteria cannot fully prevent bregma misidentification in some cases. Therefore, in cases where bregma misidentification occurs, implementing the strategy for adjusting electrode position can partially mitigate the impact of bregma misidentification on the accuracy of electrode implantation, thereby improving the accuracy of the coordinates.

### Strategy for adjusting electrode position

Among the successful adjustment cases, 88% of the rats underwent a single adjustment by placing the electrode 0.5 mm more posterior to the first implantation. It indicates that (1) this adjustment strategy is effective and feasible in addressing the issue of electrode misplacement. (2) This adjustment strategy is safe, resulting in no more than two trajectories in most cases, with no significant damage to brain tissue observed in H&E-stained slices. (3) The accuracy of averaged bregma-interaural-based approach is high in ML axis. Nevertheless, adjustment in ML axis remains advisable when the first adjustment is unsuccessful, as we demonstrated that in that case the electrode could be successfully implanted into the STN after adjusting the ML coordinate.

The electrode was adjusted 0.5 mm posterior to AP_bregma-interaural_ if the STN-like firing pattern was not detected. This adjustment strategy was informed by the results presented in Table 1. Therefore, it can help the researchers to gain a clear understanding of the AP deviation of electrode implantation caused by averaged bregma-interaural-based approach. In this study, researchers found that the averaged bregma-interaural-based approach causes an 80% likelihood of implanting the electrode 0.635 mm anterior to the intended site. Thus, adjusting the electrode 0.5 mm posterior to AP_bregma-interaural_ is a proper adjustment in case of misplacement. Additionally, the placement deviation is caused not only by bregma-interaural approach per se but also by the investigators’’habits in stereotactic surgery and the accuracy of the stereotactic apparatus. As such, we recommend that investigators conduct some pilot implantations using the averaged bregma-interaural-based approach and assess the AP deviation in their own labs. This practice allows the investigators to learn and quantify the placement deviations caused by the bregma-interaural approach. By doing so, they can develop a tailored strategy for adjusting electrode positions.

### The electrophysiology of STN

Electrophysiology plays a crucial role in BITE. In this study, the implanted electrode may typically pass through ZI before reaching STN. ZI is characterized electrophysiologically by near-silent neural activity, whereas the STN is distinguished by a burst-firing pattern with high-amplitude (0.5–1 μV), fast-bursty, and irregular spikes (Fluri et al., 2015; Deng et al., 2025). By halting the implantation immediately upon detecting signals indicative of the STN, we found that the electrode tip was positioned in dSTN in 50% of the rats, demonstrating the potential feasibility of using BITE for precise targeting in dSTN. Practically, electrodes were intended to be implanted into the STN in this study, and our electrophysiology-based method for determining the DV position of the electrode (Step 1.7) was inclined toward the dorsal STN, as validated previously. This finding is noteworthy, as the dSTN is frequently studied as a key structure in the treatment of Parkinson's disease (Herzog et al., 2004). Moving forward, the ventral STN could be targeted by advancing the electrode when the STN-like firing pattern disappears, which could also theoretically allow for targeting the middle STN. This approach provides greater flexibility for STN targeting. However, although promising in theory, further studies are needed to confirm this strategy. Additionally, we found that average STN firing rate was 51 ± 8 spk/s, which is inconsistent with the findings from previous studies (Kreiss et al., 1996; Winstanley et al., 2005). This discrepancy may be attributed to the different anesthetics employed. In our study, we used a mixture of ketamine and medetomidine, whereas halothane and chloral hydrate were used in previous cited studies. Furthermore, the position of electrode in STN was verified histologically in all rats (Fig. 6). Therefore, we recommend cautiously using firing rate as an indicator of STN if the anesthetic method is changed in the study.

Additionally, since this study was performed in healthy rats, our approach may carry the risk of bias if electrode repositioning is guided by an assessment of “typical” STN firing patterns in disease models or other genotypes, where STN activity may differ. To minimize this risk, we recommend that researchers first characterize the STN electrophysiological signatures in the specific disease model or genotype of interest before applying BITE for precise targeting. In addition, detailed characterization of STN firing features, including firing rate, amplitude, and burst duration, is warranted. Such physiological parameters may provide useful reference points for subjectively assessing accurate STN targeting.

### Calculation of the difference thresholds

In this study, we set difference thresholds for absolute values of difference between bregma-based and interaural-based STN coordinates, using the data referenced from “Rat Brain in Stereotaxic Coordinates.” The maximum dimensions of STN are ∼0.5 mm in DV axis, 1.2 mm in ML axis, and 1.1 mm in AP axis(George Paxinos, 2007). Thus, it is reasonable to set the difference thresholds for the DV, ML, and AP axes below these respective values. To ensure the accuracy of the coordinates, we opted for more stringent (lower) difference thresholds. While further lowering these difference thresholds potentially increases the accuracy of STN coordinates, achieving such more stringent thresholds poses significant challenges and may even be impractical. In this study, for all rats where the electrode was successfully implanted in STN, the differences were mostly below the difference thresholds (Table 2). However, electrode misplacement occurred in two rats, where either |AP_bregma_ − AP_interaural_| or |ML_bregma_ − ML_interaural_| exceeded twice the thresholds. Therefore, if |AP_bregma_ − AP_interaural_| or |ML_bregma_ − ML_interaural_| is more than twice the predefined thresholds, the electrode position adjustment may not be recommended, as the success rate of electrode implantation becomes low.

### Conclusion

Compared with traditional approaches, BITE provides superior precision in STN electrode implantation.

## Synthesis

Reviewing Editor: Adrien Peyrache, McGill University Decisions are customarily a result of the Reviewing Editor and the peer reviewers coming together and discussing their recommendations until a consensus is reached. When revisions are invited, a fact-based synthesis statement explaining their decision and outlining what is needed to prepare a revision will be listed below. The following reviewer(s) agreed to reveal their identity: Austen Milnerwood, Henry Denny.

This study presents and validates a novel electrophysiologically guided method (BITE) for implanting electrodes into the subthalamic nucleus (STN) in rats. It directly compares this method to two widely used stereotaxic approaches based on anatomical landmarks, one using bregma alone and the other using both bregma and interaural references. The results show a substantial increase in targeting accuracy: about five-fold improvement over the bregma-based method and two-fold over the bregma + interaural approach.

The work was reviewed by two Reviewers. They both agree that the method is innovative, addresses an important technical challenge, and is likely to improve experimental yield while reducing the number of animals used. They found the study to be well designed, statistically rigorous, and clearly described overall, with enough detail for replication. However, they identified several points that require clarification or additional analysis to strengthen the manuscript.

1) The Reviewers noted that the electrophysiological approach could be influenced by differences in neuronal excitability or firing patterns across genotypes or disease models (e.g. Parkinson's). This could lead an experimenter to reposition electrodes to obtain "typical" neuronal responses, potentially introducing bias. This limitation should be explicitly discussed.

2) The success rates reported for the bregma (17%) and bregma + interaural (40%) approaches appear low, but it is unclear how success was defined. The reviewers asked whether success refers to hitting an exact set of coordinates or simply being located within the STN. This needs to be clarified to contextualize the reported success rates.

3) The procedure for verifying electrode tip positions requires more explanation. In the Standardized protocol for electrode implantation in STN (points 1.4 and 1.5), the STN is described as a point defined by three coordinates rather than as a broader region. However, in practice, simply being within the STN may be sufficient for most experimental purposes without necessarily reaching the exact coordinate point. The difference in success rates between the two methods is striking, but depending on how "failure" is defined, many of these placements might still be considered acceptable in practice. This should be expanded upon.

4) Similarly, this raises the question of how "precise location" is determined histologically. The description provided is not entirely clear. Is verification based on the number of 10 μm sections examined and their comparison with images from Rat Brain in Stereotaxic Coordinates? On p. 7, it is stated that "targeting accuracy of both approaches was histologically verified through hematoxylin and eosin (H&E) staining to confirm electrode tip position," but this does not explain how the exact position was determined. The text also mentions that "we verified the exact electrode implantation sites using H&E staining and quantified the AP deviation by measuring the AP distance between the actual implantation site and the intended implantation site (3.7 mm posterior to bregma)." However, since bregma is not visible in histological slices, how is the "intended implantation site" inferred? Is a reference atlas or morphological mapping procedure used to estimate coordinates in tissue sections? If so, this needs to be described in detail. The subsection "Verification of electrode tip position" (p. 12) requires a much clearer explanation of the methodology.

5) The study claims that the operator followed established protocols without a learning curve, but this should be supported by data. Reviewers suggested showing that performance did not improve over time, for example with a logistic regression of success over trial order or a chi-square test comparing early vs late attempts.

6) The methods should describe how neural spike rates were calculated and how spectrograms were obtained, as this information is currently missing.

7) Visualization of data would be a welcome addition. For example, Table 1, Table 2, and animal weights could all be presented as violin plots, which would give us a much better understanding of the distribution of the data.

8) Figure 5 refers to the Zona Incerta (ZI). Figure 6 refers to the IC. Only IC is labelled in anatomical figures. Labels should correspond to exact outlined regions of interest in the images. In the text body, some introduction should be given to each region, explaining their relationship to one another, and relevance to the study.

9) Figure 6 only requires 1 example from STN and ZI so that the difference is immediately apparent to readers.

10) Section 1.6 mentions DVbregma and DVinteraural but does not explain how they were measured or used. Reviewers asked for a clear description of what is being measured and how these coordinates are compared or converted.

11) Section 1.3: "Skull drilling process generates high-frequency vibrations in the skull, which causes minor to significant bregma shift." The following claim is not substantiated by citation, nor data. An elaboration on how this happens and how this is known would be a welcome addition, especially for other surgeons in the field.

12) Section 1.3 (p. 10): The text states, "Such shift may significantly compromise the accuracy of the STN coordinates if skull drilling is executed after determining them in step 1.4 ('calculation of STN coordinates based on bregma')." while the calculation of STN coordinates based on bregma is actually described in step 1.5, not 1.4.

13) Page 19: The reference to Rat Brain in Stereotaxic Coordinates should be properly cited.

14) The subsection Verification of electrode tip position (p. 12) requires a much clearer explanation of the methodology.

15) Results, Experiment 3: It should be specified whether the dorsal and ventral sites were explicitly targeted or were simply reached while aiming for the standard coordinates while aiming for the standard three-coordinate target (determined by either the bregma- or bregma-interaural-based method). Clarification is needed to avoid ambiguity.

## Author Response

Dear Reviewers, We sincerely appreciate the reviewers' time and insightful comments, which have significantly strengthened our manuscript. Their feedbacks have enhanced the clarity, scientific rigour, and overall impact of our work. In response to the reviewers' key concerns, we have conducted a major revision of the original manuscript. Below, we provide detailed point-by-point responses to each comment along with corresponding revisions in the manuscript.

1) The Reviewers noted that the electrophysiological approach could be influenced by differences in neuronal excitability or firing patterns across genotypes or disease models (e.g. Parkinson's). This could lead an experimenter to reposition electrodes to obtain "typical" neuronal responses, potentially introducing bias. This limitation should be explicitly discussed.

Reply: We appreciate the reviewers' comment and agree that this experimenter bias during electrode positioning may happen. To mitigate this bias, we have discussed this limitation in the revised manuscript, "since this study was performed in healthy rats, our approach may carry the risk of bias if electrode repositioning is guided by an assessment of 'typical' STN firing patterns in disease models or other genotypes, where STN activity may differ. To minimize this risk, we recommend that researchers first characterize the STN electrophysiological signatures in the specific disease model or genotype of interest before applying BITE for precise targeting. In addition, detailed characterization of STN firing features, including firing rate, amplitude, and burst duration, is warranted. Such physiological parameters may provide useful reference points for subjectively assessing accurate STN targeting." 2) The success rates reported for the bregma (17%) and bregma + interaural (40%) approaches appear low, but it is unclear how success was defined. The reviewers asked whether success refers to hitting an exact set of coordinates or simply being located within the STN. This needs to be clarified to contextualize the reported success rates.

Reply: We thank the reviewers for this clarification. To avoid ambiguity, we have revised the manuscript to state: "An implantation of the electrode was considered a "success" if the electrode tip was located anywhere within the anatomical boundaries of the STN, regardless of whether it matched the intended coordinates." 3) The procedure for verifying electrode tip positions requires more explanation. In the Standardized protocol for electrode implantation in STN (points 1.4 and 1.5), the STN is described as a point defined by three coordinates rather than as a broader region. However, in practice, simply being within the STN may be sufficient for most experimental purposes without necessarily reaching the exact coordinate point. The difference in success rates between the two methods is striking, but depending on how "failure" is defined, many of these placements might still be considered acceptable in practice. This should be expanded upon.

Reply: We thank the reviewers' comment, and we agree that "in practice, simply being within the STN may be sufficient for most experimental purposes without necessarily reaching the exact coordinate point." Thus, we revised the manuscript to state "An implantation of the electrode was considered a "success" if the electrode tip was located anywhere within the anatomical boundaries of the STN, regardless of whether it matched the intended coordinates".

4) Similarly, this raises the question of how "precise location" is determined histologically. The description provided is not entirely clear. Is verification based on the number of 10 μm sections examined and their comparison with images from Rat Brain in Stereotaxic Coordinates? Reply: We thank the reviewers for this comment. To avoid ambiguity, we have revised the manuscript to state: "The electrode tip position was confirmed through histological analysis. Following perfusion with phosphate-buffered saline and 4% paraformaldehyde, the brain was extracted, embedded in paraffin, and coronally sectioned at a thickness of 10 µm. Every section containing the visible electrode track was collected and stained with H&E. The precise location of the electrode tip was determined by aligning the histological sections with the corresponding sections in the Paxinos and Watson rat brain atlas (George Paxinos, 2007). This alignment was performed using some anatomical landmarks visible in both the H&E-stained sections and the atlas, including the third ventricles and the shape of hippocampus, optic fiber. Additionally, the H&E stained STN is in dark purple with densely packed neurons and is clearly delineated from the surrounding zona incerta and internal capsule (see Figure 5). These features facilitate the precise determination of the electrode tip location within STN when implantation is successful. An implantation of the electrode was considered a "success" if the electrode tip was located anywhere within the anatomical boundaries of the STN, regardless of whether it matched the intended coordinates. The AP coordinate of the electrode tip was obtained from the atlas (George Paxinos, 2007). In experiment 2, this AP coordinate was used to calculate the AP deviation as the absolute difference between the actual and intended coordinates." 5) On p. 7, it is stated that "targeting accuracy of both approaches was histologically verified through hematoxylin and eosin (H&E) staining to confirm electrode tip position," but this does not explain how the exact position was determined.

Reply: We agreed on this point. To avoid ambiguity, we added the explanation on the "Verification of electrode tip position" section that "...Every section containing the visible electrode track was collected and stained with H&E. The precise location of the electrode tip was determined by aligning the histological sections with the corresponding sections in the Paxinos and Watson rat brain atlas (George Paxinos, 2007). This alignment was performed using some anatomical landmarks visible in both the H&E-stained sections and the atlas, including the third ventricles and the shape of hippocampus, optic fiber. Additionally, the H&E stained STN is in dark purple with densely packed neurons and is clearly delineated from the surrounding zona incerta and internal capsule (see Figure 5). These features facilitate the precise determination of the electrode tip location when implantation within the STN is successful. An implantation of the electrode was considered a "success" if the electrode tip was located anywhere within the anatomical boundaries of the STN, regardless of whether it matched the intended coordinates ..." 6) The text also mentions that "we verified the exact electrode implantation sites using H&E staining and quantified the AP deviation by measuring the AP distance between the actual implantation site and the intended implantation site (3.7 mm posterior to bregma)." However, since bregma is not visible in histological slices, how is the "intended implantation site" inferred? Is a reference atlas or morphological mapping procedure used to estimate coordinates in tissue sections? If so, this needs to be described in detail. The subsection "Verification of electrode tip position" (p. 12) requires a much clearer explanation of the methodology.

Reply: Thank you for this comment. The intended implantation site was defined as 3.7 mm posterior to bregma. Bregma was visible and identified on the skull prior to electrode implantation, and the electrode was positioned accordingly (details please refer to the point 1.2 and 1.3 ). To clarify this point, we revised the manuscript to state "To achieve this goal, we verified the exact electrode implantation sites using H&E staining and quantified the AP deviation by measuring the AP distance between the actual implantation site and the intended implantation site (3.7 mm posterior to bregma). The actual implantation site in AP axis was determined by reference to the Paxinos and Watson atlas(George Paxinos, 2007)." Additionally, we revised the "Verification of electrode tip position" to "The electrode tip position was confirmed through histological analysis. Following perfusion with phosphate-buffered saline and 4% paraformaldehyde, the brain was extracted, embedded in paraffin, and coronally sectioned at a thickness of 10 µm. Every section containing the visible electrode track was collected and stained with H&E. The precise location of the electrode tip was determined by aligning the histological sections with the corresponding sections in the Paxinos and Watson rat brain atlas (George Paxinos, 2007). This alignment was performed using some anatomical landmarks visible in both the H&E-stained sections and the atlas, including the third ventricles and the shape of hippocampus, optic fiber. Additionally, the H&E stained STN is in dark purple with densely packed neurons and is clearly delineated from the surrounding zona incerta and internal capsule (see Figure 5). These features facilitate the precise determination of the electrode tip location within STN when implantation is successful. An implantation of the electrode was considered a "success" if the electrode tip was located anywhere within the anatomical boundaries of the STN, regardless of whether it matched the intended coordinates. The AP coordinate of the electrode tip was obtained from the atlas (George Paxinos, 2007). In experiment 2, this AP coordinate was used to calculate the AP deviation as the absolute difference between the actual and intended coordinates." 7) The study claims that the operator followed established protocols without a learning curve, but this should be supported by data. Reviewers suggested showing that performance did not improve over time, for example with a logistic regression of success over trial order or a chi-square test comparing early vs late attempts.

Reply: Thank you for this comment. To be more rigorous, we revised the manuscript to state: "Statistical analysis Success rate dynamics: To assess potential learning curve effects, we compared early versus later success rates for bregma-based approach and averaged bregma-interaural based approach. For the bregma-based method (2022), success rates were compared between the first and second six months. For the averaged bregma-interaural method (2023), we made the same comparison. Before analysis, expected cell frequencies in all contingency tables were checked: Fisher's exact test was used when any expected count was <5, otherwise Pearson's chi-square test was applied.

Results Success rate dynamics There was no significant difference in the success rate of the bregma-based approach between the first and second six-month periods. (0% vs 33%; p=0.206; Fisher's exact test). Similarly, the success rate of the averaged bregma-interaural approach did not differ significantly between the first and second six-month periods (44% vs 30%; p=0.449; Fisher's exact test)." 8) The methods should describe how neural spike rates were calculated and how spectrograms were obtained, as this information is currently missing.

Reply: Thank you for this important comment. To clarify, we revised the manuscript to state "Power Spectral Density We aimed to investigate the STN oscillations. To achieve this, we analyzed STN oscillations using spectral analysis. Raw data were collected at 30 kHz. Power spectral density was computed via short-time Fourier transforms with standard parameters (Hanning window, 50% overlap). This approach follows the methodology detailed in Deng et al (Deng et al., 2025).

Firing rate calculation Raw signals were band-pass filtered (300-3000 Hz) to isolate spikes. Spike detection was performed using a threshold based on the median absolute deviation, followed by template matching to refine spike selection. Spike times were used to calculate the spike count and firing rate (spikes per second)." 9) Visualization of data would be a welcome addition. For example, Table 1, Table 2, and animal weights could all be presented as violin plots, which would give us a much better understanding of the distribution of the data.

Reply: Thank you for this comment. To improve visualization, we have added violin plots to the manuscript (see Figure 6).

10) Figure 5 refers to the Zona Incerta (ZI). Figure 6 refers to the IC. Only IC is labelled in anatomical figures. Labels should correspond to exact outlined regions of interest in the images. In the text body, some introduction should be given to each region, explaining their relationship to one another, and relevance to the study.

Replay: We thank you for this important comment. To clarify, we revised the corresponding figures (see Figure 5 and Figure 7) to make it more clearer to the readers.

11) Figure 6 only requires 1 example from STN and ZI so that the difference is immediately apparent to readers.

Replay: Initially, we intended to present a single example from the STN and ZI to illustrate this result. However, we decided it is more informative to show all the data collected in this study, which not only ensures transparency for the readers but also fully addresses any doubts regarding the histology results.

12) Section 1.6 mentions DVbregma and DVinteraural but does not explain how they were measured or used. Reviewers asked for a clear description of what is being measured and how these coordinates are compared or converted.

Reply: Thank you for this comment. To improve clarity, we revised the manuscript to state "Using a micromanipulator and surgical microscope, we adjusted the electrode position until the tip was precisely aligned with the ear bar tip (see figure. 4A-C), the scales on the DV, ML, and AP arms were then recorded as AP', ML', and DV', representing the coordinates of the interaural zero. The STN coordinates based on inter-aural zero were measured as 5.3 mm anterior (APinteraural = AP'+ 5.3), 2.4 mm lateral for the right STN (MLinteraural = ML'- 2.4), and 1.65 mm superior (DVinteraural = DV'+ 1.65) to interaural zero, respectively (George Paxinos, 2007).

1.5 Calculations of STN coordinates based on bregma Using a surgical microscope, we carefully aligned the electrode as close as possible to the skull surface at bregma and recorded the scales as AP', ML' and DV' on the respective arms. According to the atlas (George Paxinos, 2007) and the previous experiences in targeting STN (Baunez and Robbins, 1997; Baunez et al., 2001; Winstanley et al., 2005; Siegle et al., 2017; Pelloux et al., 2018), the STN coordinates relative to bregma were determined as 3.7 mm posterior (APbregma=AP'- 3.7), 2.4 mm lateral for the right STN (MLbregma=ML'- 2.4), and 8.35mm ventral (DVbregma=DV'- 8.35).

1.6 Calculations of STN coordinates based on averaged bregma-interaural based approach.

We calculated the absolute values of difference between bregma based and interaural based STN coordinates as |APbregma - APinteraural|, |MLbregma - MLinteraural|, and |DVbregma - DVinteraural|. The difference thresholds were set to |APbregma - APinteraural| {less than or equal to} 0.5 mm, |MLbregma - MLinteraural| {less than or equal to} 0.3 mm, and |DVbregma - DVinteraural| {less than or equal to} 0.3 mm. If the calculated absolute values of difference were within the difference thresholds, we averaged the bregma-based and interaural-zero based coordinates to obtain the final STN coordinates: APbregma-interaural = (APbregma - APinteraural)/2, MLbregma-interaural = (MLbregma - MLinteraural)/2 and DVbregma-interaural = (DVbregma - DVinteraural)/2. If any differences exceeded the difference thresholds, we used the bregma-based coordinates for AP, ML or DV. " 13) Section 1.3: "Skull drilling process generates high-frequency vibrations in the skull, which causes minor to significant bregma shift." The following claim is not substantiated by citation, nor data. An elaboration on how this happens and how this is known would be a welcome addition, especially for other surgeons in the field.

Reply: Thank you for this comment. To enhance the clarity, we revised the manuscript to state, "The skull drilling process generates high-frequency vibrations and mechanical forces. We hypothesize that these forces could cause minor shift of the skull, potentially leading to a bregma shift. Although we have not yet quantified this phenomenon in this setup, we occasionally observed indications of such shifts during surgery. Thus, we recommend that operators perform the critical step of coordinate calculation (Step 1.5) after any major drilling to preemptively mitigate this potential source of error. Future work is needed to quantify this phenomenon." 14) Section 1.3 (p. 10): The text states, "Such shift may significantly compromise the accuracy of the STN coordinates if skull drilling is executed after determining them in step 1.4 ('calculation of STN coordinates based on bregma')." while the calculation of STN coordinates based on bregma is actually described in step 1.5, not 1.4.

Reply: Thank you for pointing out this mistake. We have revised this statement to "The skull drilling process generates high-frequency vibrations and mechanical forces. We hypothesize that these forces could cause minor shift of the skull, potentially leading to a bregma shift. Although we have not yet quantified this phenomenon, we occasionally observed indications of such shifts during surgery. Thus, we recommend that operators perform the critical step of coordinate calculation (Step 1.5) after any major drilling to preemptively mitigate this potential source of error. Future work is needed to quantify this phenomenon." in the manuscript.

15) Page 19: The reference to Rat Brain in Stereotaxic Coordinates should be properly cited.

Reply: Thank you for this comment. We revised this citation to "George Paxinos CW (2007) The Rat Brain in Stereotaxic Coordinates: Academic Press." 16) The subsection Verification of electrode tip position (p. 12) requires a much clearer explanation of the methodology.

Reply: Thank you for this comment. To enhance the clarity, we rewrote this section, "The electrode tip position was confirmed through histological analysis. Following perfusion with phosphate-buffered saline and 4% paraformaldehyde, the brain was extracted, embedded in paraffin, and coronally sectioned at a thickness of 10 µm. Every section containing the visible electrode track was collected and stained with H&E. The precise location of the electrode tip was determined by aligning the histological sections with the corresponding sections in the Paxinos and Watson rat brain atlas (George Paxinos, 2007). This alignment was performed using some anatomical landmarks visible in both the H&E-stained sections and the atlas, including the third ventricles and the shape of hippocampus, optic fiber. Additionally, the H&E stained STN is in dark purple with densely packed neurons and is clearly delineated from the surrounding zona incerta and internal capsule (see Figure 5). These features facilitate the precise determination of the electrode tip location within STN when implantation is successful. An implantation of the electrode was considered a "success" if the electrode tip was located anywhere within the anatomical boundaries of the STN, regardless of whether it matched the intended coordinates. The AP coordinate of the electrode tip was obtained from the atlas (George Paxinos, 2007). In experiment 2, this AP coordinate was used to calculate the AP deviation as the absolute difference between the actual and intended coordinates." 17) Results, Experiment 3: It should be specified whether the dorsal and ventral sites were explicitly targeted or were simply reached while aiming for the standard coordinates while aiming for the standard three-coordinate target (determined by either the bregma- or bregma-interaural-based method). Clarification is needed to avoid ambiguity.

Reply: Thank you for this comment. The electrodes were intended for general STN implantation, and our electrophysiology-based method for determining dorsoventral position (Step 1.7) is biased toward the dorsal STN. Specifically, we revised the manuscript to "...Electrophysiology plays a crucial role in BITE. In this study, the implanted electrode may typically pass through ZI before reaching STN. ZI is characterized electrophysiologically by near-silent neural activity, whereas the STN is distinguished by a burst-firing pattern with high-amplitude (0.5-1 μV), fast-bursty, and irregular spikes(Fluri et al., 2015; Deng et al., 2025). By halting the implantation immediately upon detecting signals indicative of the STN, we found that the electrode tip was positioned in dSTN in 50% of the rats, demonstrating the potential feasibility of using BITE for precise targeting in dSTN. Practically, electrodes were intended to be implanted into the STN in this study, and our electrophysiology-based method for determining the DV position of the electrode (step 1.7) was inclined toward the dorsal STN, as validated previously. This finding is noteworthy, as the dSTN is frequently studied as a key structure in the treatment of Parkinson's disease(Herzog et al., 2004). Moving forward, the ventral STN could be targeted by advancing the electrode when the STN-like firing pattern disappears, which could also theoretically allow for targeting the middle STN. This approach provides greater flexibility for STN targeting. However, although promising in theory, further studies are needed to confirm this strategy. ..."
